# A Blood Change in Gas Poisoning

**Published:** 1918

**Authors:** 


***A Blood Change in Gas Poisoning.*** By JAMES MILLER, Capt. R. A. M. C. (T.). Abs. from the *Lancet*, May 26, 1917.

The type of gas poisoning and the chemical nature of gas appears to make no difference in determining blood changes.

Although there is generally an increase in lymphocytes in cases with persistent symptoms, the author states that he has observed cases of moderately severe gassing in which the count remains either normal or within the limits of error.

In order that the test may be of value the author considers that the lymphocytosis should be so marked that the lymphocyte count approaches closely that of the polymorphonuclear leucocytes. If this is the case, it may be assumed that the patient is still suffering from gassing.

The ordinary small lymphocyte of the blood is the cell that is increased. The increase is an absolute one and may be accentuated or the reverse in a differential estimate as the leucocyte count may for any reason be increased or diminished.

The author states that the cause of the lymphocytosis is not clear, but he thinks it may be in part due to a chronic inflammatory change in the respiratory and gastric mucous membranes.

